# Fine-tuning of post-weaning pig microbiome structure and functionality by in-feed zinc oxide and antibiotics use

**DOI:** 10.3389/fcimb.2024.1354449

**Published:** 2024-02-07

**Authors:** Juan M. Ortiz Sanjuán, Edgar G. Manzanilla, Raúl Cabrera-Rubio, Fiona Crispie, Paul D. Cotter, Juan J. Garrido, Daniel Ekhlas, Lorcan O’Neill, Héctor Argüello

**Affiliations:** ^1^ Pig Development Department, Teagasc Grassland Research and Innovation Centre, Moorepark, Cork, Ireland; ^2^ Grupo de Genómica y Mejora Animal, Departamento de Genética, Facultad de Veterinaria, Universidad de Córdoba, Córdoba, Spain; ^3^ School of Veterinary Medicine, University College Dublin, Dublin, Ireland; ^4^ Teagasc Food Research Centre, Moorepark, Cork, Ireland; ^5^ APC Microbiome Institute Science Foundation Ireland (SFI) Research Centre, University College Cork, Cork, Ireland; ^6^ VistaMilk Science Foundation Ireland (SFI) Research Centre, Cork, Ireland; ^7^ Departamento de Sanidad Animal, Facultad de Veterinaria, Universidad de León, León, Spain

**Keywords:** antimicrobial, environment, microbiome, piglets, post-weaning diarrhea, weaning, zinc oxide

## Abstract

**Introduction:**

Post-weaning diarrhoea (PWD) is a multifactorial disease that affects piglets after weaning, contributing to productive and economic losses. Its control includes the use of in-feed prophylactic antibiotics and therapeutic zinc oxide (ZnO), treatments that, since 2022, are no longer permitted in the European Union due to spread of antimicrobial resistance genes and pollution of soil with heavy metals. A dysbiosis in the microbiota has been suggested as a potential risk factor of PWD onset. Understanding pig’s microbiota development around weaning and its changes in response to ZnO and antibiotics is crucial to develop feasible alternatives to prophylactic and metaphylactic antimicrobial use.

**Methods:**

This study used shotgun metagenomic sequencing to investigate the environmental and faecal microbiota on 10 farms using (Treated) or not using (ZnO-free) in-feed antibiotics and ZnO during the first 14 days post-weaning (dpw). Environmental samples from clean pens were collected at weaning day (0dpw), and faecal samples at 0, 7 and 14dpw. Diarrhoeic faecal samples were collected at 7dpw when available.

**Results:**

The analysis of data revealed that the faecal microbiota composition and its functionality was impacted by the sampling time point (microbiota maturation after weaning) but not by the farm environment. Treatment with antibiotics and ZnO showed no effects on diversity indices while the analyses of microbiota taxonomic and functional profiles revealed increased abundance of taxa and metabolic functions associated with *Phascolarctobacterium succinatutens* or different species of *Prevotella spp*. on the Treated farms, and with *Megasphaera elsdenii* and *Escherichia coli* on the ZnO-free farms. The analysis of diarrhoea samples revealed that the treatment favoured the microbiota transition or maturation from 0dpw to 14dpw in Treated farms, resembling the composition of healthy animals, when compared to diarrhoea from ZnO-free farms, which were linked in composition to 0dpw samples.

**Discussion:**

The results provide a comprehensive overview of the beneficial effects of ZnO and antibiotics in PWD in the microbiota transition after weaning, preventing the overgrowth of pathogens such as pathogenic *E. coli* and revealing the key aspects in microbiota maturation that antibiotics or ZnO alternatives should fulfil.

## 1 Introduction

Post weaning diarrhea (PWD) is a multifactorial disease that affects piglets at weaning period and often requires antimicrobial treatment (Luppi, 2017; Fairbrother and Nadeau, 2019). Weaning on commercial pig farms is associated with numerous challenges such as early separation from the sow, sudden transition from milk to solid feed and environmental stress. These challenges result in immunological, physiological and microbial imbalances that create a window of opportunity for enteric pathogens to cause disease (Rhouma et al., 2017; Bonetti et al., 2021). Enterotoxigenic *Escherichia coli* (ETEC) is the main etiological agent associated with PWD (Rhouma et al., 2017; Fairbrother and Nadeau, 2019). Traditionally, its prevention and control has been based on the prophylactic and metaphylactic use of in-feed antibiotics and/or therapeutic zinc oxide (ZnO) (Poulsen, 1995; Sales, 2013). In the European Union (EU), however, both practices have been restricted since 2022. Concerns over transmission of antimicrobial resistant bacteria between animals and humans led to an outright ban of prophylactic antimicrobial use (AMU) and restrictions on metaphylactic AMU in animals (European Commission, 2019a), while concerns over soil pollution with zinc (Zn) led to ban on therapeutic use of ZnO (Standing Committee on veterinary medicinal products, 2017; European Commission, 2019b). Other important pig producing countries like China or the US still allow the prophylactic use of ZnO and oral antimicrobials, although future directives to preserve antimicrobials may follow the European approach.

Controlling PWD without using ZnO or in-feed antimicrobials is challenging and new approaches to improve the gut health of piglets are needed. A better understanding of how antimicrobials and ZnO prevent PWD is needed to develop new approaches. The mechanisms of action for antibiotics are well described. However, the exact mechanism of action of ZnO in PWD control is not completely understood despite its proven efficacy. Zinc is involved in several physiological processes including digestion and immune response that impact animal performance (Sales, 2013; Bonetti et al., 2021). Zinc participates as a co-factor for multiple enzymes and is required for multiple biochemical reactions in both eukaryote and prokaryote organisms (Wątły et al., 2016; Sloup et al., 2017). In addition, zinc exhibits antimicrobial activity against certain groups of bacteria (Söderberg et al., 1990; Pasquet et al., 2014) and modifies the intestinal microbiota (Li et al., 2020; Pieper et al., 2020). This intestinal microbiota provides colonization resistance against enteric pathogens whereby beneficial microorganisms occupy niches, compete for substrates or produce inhibitory molecules such as bacteriocins (Spees et al., 2013; Dou et al., 2017). Despite the existing information from previous studies, we are still far from understanding the effects of the regular use of antimicrobials and ZnO on the intestinal microbiota of the pigs and on the environmental microbiota on commercial farms. Further research in this area can provide relevant insights relating to the microbial changes that occur at and after weaning, how antibiotic and ZnO treatments affect the intestinal microbiota and its potential impacts on PWD outcome.

The objective of this study was to describe the environmental microbiota of commercial pig farms and the intestinal microbiota (composition and functionality) of pigs in the first 2 weeks post weaning including both farms using in-feed prophylactic antibiotic and ZnO (Treated) and farms free of these treatments (ZnO-free). The results will provide a reference for future studies about pig farming within a context of antibiotic and ZnO limited use at weaning.

## 2 Materials and methods

### 2.1 Sampling

This study was licensed by Teagasc Animal Ethics Committee and was carried out on commercial pig farms in the Republic of Ireland. Ten farms were selected, five farms with routine use of in-feed prophylactic antibiotics and therapeutic ZnO (3,000 ppm) during first two weeks post-weaning (Treated, n = 5) and 5 farms that had not used either strategy during the previous three years (ZnO-free, n = 5). The farms used in this study ranged in size a size between 200 and 3000 sows, were all farrow-to-finish operations, and weaned piglets between 28 and 32 days of age. The antibiotics used on the Treated farms were amoxicillin (Stabox, Virbac, France) or trimethoprim and sulfadiazine in combination (Sulfoprim, Univet Limited, Ireland) at the dose indicated by the manufacturer. On each farm, two pens from two different rooms were sampled. Pen size varied depending on the farm and ranged between 12 and 72 pigs per pen with similar stocking densities, slightly above legal requirement, throughout. Environmental sampling was performed in empty clean pens immediately before the pigs were moved into the rooms on weaning day (0 day post-weaning, dpw) using sponge swabs (3M™ Sponge-Stick in sample bag with 10 mL D/E neutralizing broth, 3M Deutschland GmbH, Neuss, Germany). One swab was used to sample the feeders and the drinkers in the pen (FD) and another swab was used to sample two sections of 50 cm2 of the walls and the floor of the pen (WF). Pig fecal samples were collected at 0, 7 and 14dpw. In addition, a diarrheic fecal sample was collected at 7dpw if available. For the fecal sampling, one random freshly voided fecal sample from one pig per pen was collected and transferred to 1.5mL microcentrifuge tube. Each sample was collected using a sterile 140x7mm conical steel spatula avoiding the part in direct contact with the floor. Samples were transported to the laboratory under cooling conditions (in less than 2 hours) where swabs were processed extracting the sampled material from the swabs using 5 mL of sterile Phosphate Saline Buffer (PBS) 1X. Approximately 8 mL and 16 mL were recovered from each WF and FD swab, respectively, and transferred to a 20mL centrifuge tube. These tubes were centrifuged at 3000 x g for 15 min at 4° C, the supernatant was discarded, and the pellet was suspended in 1mL of PBS and transferred to a 1.5 mL tube. The processed environmental samples and the fecal samples were stored at -80° C until DNA extraction.

### 2.2 DNA extraction and library preparation

The DNA was extracted using the QIAamp PowerFecal Pro DNA Kit (Qiagen, Crawley, West Sussex, UK) following the manufacturer’s instructions, using 200 ± 50 mg of fecal content from samples. Environmental samples were previously thawed on ice, centrifuged at 15000 rpm for 1 min at 4° C, the supernatant was discarded and pellet was used for DNA extraction. A Qubit fluorometer (Qubit 3, BioSciences, Dublin, Ireland) was used to determine the total DNA concentration. The 2 samples from the different rooms for each type and time point were pooled by adding 5µL of each sample at a concentration of 1ng/µL. Paired-end sequencing libraries were prepared from the extracted DNA using the Illumina Nextera XT Library Preparation Kit (Illumina Inc., San Diego, CA) followed by sequencing on the Illumina NextSeq 500 platform using high-output chemistry (2 × 150 bp) according to the manufacturer’s instructions. Library size from each sample was assessed on an Agilent Technology 21000 Bioanalyzer using a High Sensitivity DNA chip.

### 2.3 Bioinformatic analysis

Raw reads were filtered using trimmomatic v0.38 (Bolger et al., 2014). An average quality threshold score of 15 in a sliding window of 4 base pairs was used to trim reads below the threshold. A minimum length of 40 base pairs was ensured for all reads. Bowtie2 v2.4.4 (Langmead and Salzberg, 2012) was used to map the reads against host and human reference genomes, keeping the unmapped reads for the downstream analysis. Reference genomes were downloaded from Illumina iGenomes (https://support.illumina.com/sequencing/sequencing_software/igenome.html). Read duplicates were removed using the clumpify.sh tool in bbmap 38.22 (Bushnell, 2014). Analysis of microbial composition was carried out using Metaphlan v3.0 (Beghini et al., 2021). Functional profiles were assigned using HUMAnN v3.0 (Beghini et al., 2021). Gene families identified by HUMAnN were regrouped in MetaCyc metabolic reactions and Pfam protein domains functional categories using the utility script ‘humann_regroup_table’. MetaCyc is a database of metabolic pathways from all domains of life, containing pathways involved in primary and secondary metabolism, associated metabolites, reactions, enzymes, and genes (Caspi et al., 2020). Pfam is a comprehensive Database of Protein Domain Families (Finn et al., 2010). Abundance of MetaCyc metabolic reactions and Pfam protein domains was obtained from UniRef90 gene families using HUMAnN v3.0. Processed reads were assembled into contigs using the metaSPAdes pipeline from SPAdes v3.15.3 (Nurk et al., 2017). Mass screening of assembled contigs for *E. coli* virulence factors was performed with ABRicate (v1.0.1; https://github.com/tseemann/abricate), using the Ecoli_VF database (https://github.com/phac-nml/ecoli_vf).

### 2.4 Statistical analysis

All analyses were carried out in R v4.0.2 (R Core Team, 2022) with alpha level for significance of 0.05 and trend between 0.05 and 0.10 unless otherwise indicated. The fixed factors to be studied were the type of sample (FD, WF, feces or diarrhea), treatment or not with in-feed ZnO and antibiotics (Treated or ZnO-free) and day post weaning (0dpw, 7dpw or 14dpw). The type of sample and day post weaning were merged into a unique factor named “type-dpw”, having 6 different levels for this factor: feces 0dpw, feces 7dpw, feces 14dpw, diarrhea 7dpw, FD and WF. The farm was included in all the clustering analyses.

The effect of the treatment on the microbiota was studied between and within each type-dpw level. Alpha and beta diversities were computed at both the species and functional level using the R package Vegan v2.5-7 (Jari Oksanen et al., 2020). For alpha diversity estimation, Species richness, Inverse Simpson, and Shannon and Pielou evenness indices of diversity were calculated. Statistical differences in alpha diversity indexes were tested, after checking their normal distribution, with ANOVA and pairwise compared with Tukey (car v3.0.10) (Broom et al., 2006); multcompView v0.1.8, (Graves et al., 2019) and lsmeans v2.30.0 (Lenth, 2016) R packages or otherwise by using the Kruskal-Wallis test followed by pairwise Wilcoxon tests (R Core Team, 2022) R package. Beta diversity and ordination of samples were performed by non-metric multidimensional scaling (NMDS) of previously calculated Weighted Unifrac and Aitchison distances between samples by Species and functional abundance data, respectively. Weighted Unifrac distances on the species abundance table were calculated using the utility R script calculate_unifrac.R from Metaphlan. Aitchison distances were computed calculating the Euclidean distances of the CLR transformed pathways. Ordination of virulence factors data was performed by PCoA of distances calculated using Simple matching coefficient (Sokal and Michener, 1958). Separation between groups was tested with PERMANOVA (adonis2 and pairwise adonis) (Jari Oksanen et al., 2020; Martinez Arbizu, 2020). Factors and species influencing the ordination were assessed by linear models fitting on the ordination results (*envfit* function in Vegan R package). All P-values were adjusted by Benjamini-Hochberj (BH) procedure (Benjamini and Hochberg, 1995). For fitting species in ordination space, taxa and pathways were filtered, keeping the top 15 species and functions with the highest mean abundance across samples.

MetaCyc pathways obtained using HUMAnN were regrouped into MetaCyc superclasses using ‘humann2meco’ function from microeco package in R (Liu et al., 2021). Bacteria and function abundance analyses among type, dpw and treatment were performed using Linear Discriminant Analysis Effect Size (LDA LEfSE) (Segata et al., 2011). Data grouped in variables type_dpw, treatment, or type_dpw_treatment were used as classes selecting an alpha cut-off of 0.05 and a LDA threshold of 4 for type_dpw species composition analysis and type_dpw_treatment metacyc grouped superclass2 analysis, and 2 for species composition comparison between treatments. Species and functional genes explaining differences between classes were determined by LEfSE using Kruskal-Wallis test (P < 0.05) followed by linear discriminant analysis. Plots were built using ggplot2 v3.3.3 and pheatmap v1.0.12 in R (Wickham, 2016; Raivo, 2019) and the figures produced were subsequently arranged using Inkscape software v1.0.2 (Inkscape Project, 2020).

## 3 Results

### 3.1 The porcine gut microbiota shifts during weaning and, in cases of PWD, is impacted by antibiotic and ZnO treatment

**Figure 1:**
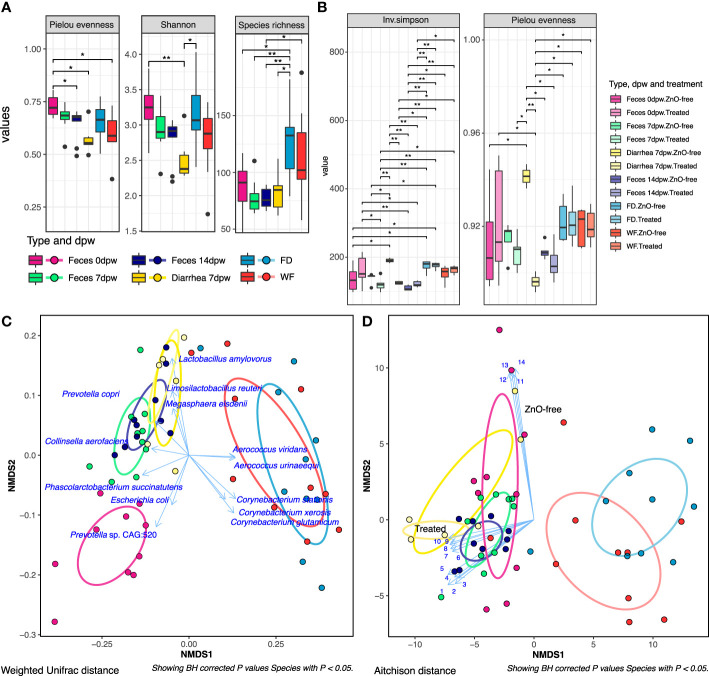
Analysis of microbiome diversity by type of sample, day post-weaning (dpw) and treatment. **(A)** Results of alpha diversity: Pielou evenness, Shannon and Species richness diversity indices at Species level. (* P < 0.05; ** P < 0.01). **(B)** Alpha diversity values by treatment, sample type and day post-weaning variables in metabolic profiles. **(C)** Non-metric multidimensional scaling (NMDS) plot visualizing between sample beta diversity of microbiota at species level. **(D)** NMDS plot visualizing between sample beta diversity of microbiota at metabolic pathways level. Blue arrows display the top 15 species and pathways with the highest mean abundance returned by “envfit” model, that significantly influenced the ordination (BH P. adjusted value, P < 0.05). The length of the arrow is proportional to the r^2^ statistic returned by the “envfit” model. Pathways fitted onto ordination are indicated as numbers, ordered according to its NMDS coordinates. 1. Nucleoside and Nucleotide Biosynthesis: *Lactobacillus amylovorus*, 2. Cell Structure Biosynthesis: *L. amylovorus*, 3. Carbohydrate Biosynthesis: *L. amylovorus*, 4. Cofactor, Carrier, and Vitamin Biosynthesis: *L. amylovorus*, 5. Amino Acid Biosynthesis: *L. amylovorus*, 6. Fatty Acid and Lipid Biosynthesis: *Limosilactobacillus reuteri*, 7. Cell Structure Biosynthesis: *L. reuteri*, 8. Amino Acid Biosynthesis: *L. reuteri*, 9. Nucleoside and Nucleotide Biosynthesis: *L. reuteri*, 10. Cofactor, Carrier, and Vitamin Biosynthesis: *L. reuteri*, 11. Amino Acid Biosynthesis: *E. coli*, 12. Fatty Acid and Lipid Biosynthesis: *E. coli*, 13. Nucleoside and Nucleotide Biosynthesis: *E. coli*, 14. Cofactor, Carrier, and Vitamin Biosynthesis: *E. coli*. Ellipses drawn on Figures **(C, D)** represent each type-dpw level, along with the Diarrhea 7dpw ZnO-free samples, with their shape being defined by the covariance within each group. Taxonomic and functional profiling of sequences was performed using Metaphlan3 and HUManN3 respectively.

Results obtained from computing richness (Species richness) and diversity (Shannon, Inverse Simpson, Pielou) did not reveal any difference by the treatment (ZnO-free vs Treated) or farm (farms 1 to 10) variables, while there were differences when data was analysed by sample-type and days post-weaning (dpw) factors. **Figure 1A** summarises the α-diversity taxonomic results while functional analyses are shown in **Figure 1B**. Fecal diversity evenness measured by Pielou index decreased from weaning (0dpw) to the last sampling performed at 14dpw (P < 0.05). Interestingly, the lowest diversity value across the three indexes analysed was obtained in Diarrhea 7dpw samples, which was lower than Feces 0dpw in both Pielou evenness and Shannon indexes (P = 0.022 and P = 0.009, respectively). Diversity values in environmental samples collected at weaning revealed a large species richness in FD and to a lower extent in WF categories. While taxonomic α-diversity was not impacted by treatment, the analysis of diversity in metabolic pathways revealed differences between Treated and ZnO-free samples that were more evident in Diarrhea 7dpw samples. These functional differences were also significant for sample-type and days post-weaning variables (**Figure 1B**).

Similar to the results observed in α-diversity, neither treatment nor farm factors impacted the β-diversity ordination of the microbiota (**Supplementary Table S1**) whereas both sample-type and days post-weaning variables contributed to microbiota spatial distribution (**Supplementary Table S2**, P < 0.05), at both species composition and metabolic pathways profiles, showing marked differences in diversity between fecal and environmental samples (**Figures 1C, D**). The ordination analyses revealed a clear separation by treatment group (Treated vs ZnO-free) in Diarrhea 7dpw samples, which were more apparent in functional than taxonomic data. The most abundant species and pathways that influenced the microbiota ordinations are shown in **Figures 1C, D**.

### 3.2 Environmental and fecal samples have different microbiota compositions at genus level and further sub-categories can be defined by species composition

**Figure 2:**
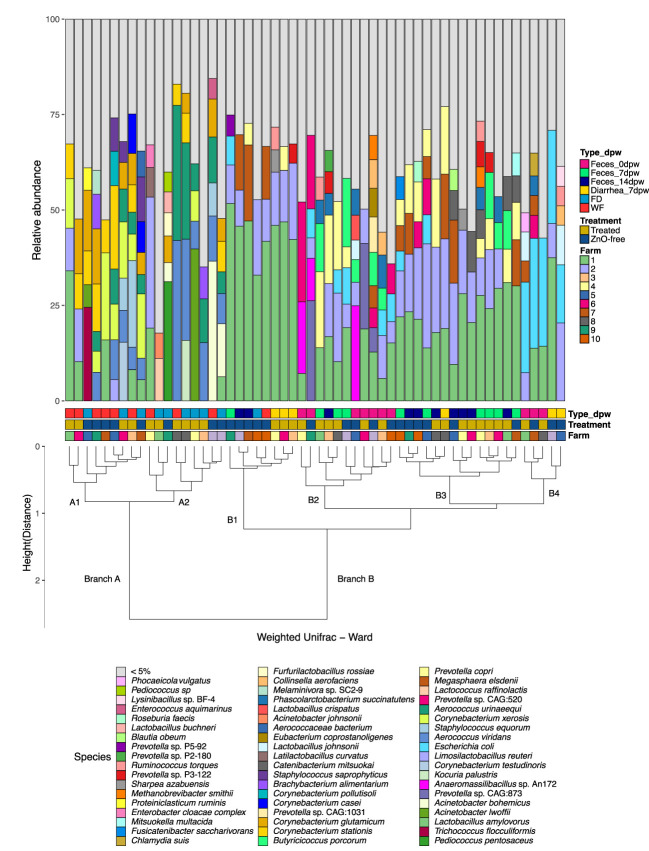
Relative abundance of the main species in each sample from 10 commercial farms. Profiles of samples are ordered by Ward clustering of the squared Weighted Unifrac distances between samples. Cluster dendrogram represents the similarity between samples regarding its microbial composition. Variables information in each sample (from lower to upper level: Farm, Treatment, Type-dpw) are indicated in the coloured squares below the bars. Taxonomic assignment was performed using Metaphlan3.

We further explored the sample metagenome structure for their similarity in microbial abundance using Ward clustering of species weighted Unifrac phylogenetic distances (**Figure 2**; **Supplementary Figure S1**). The analysis clearly split samples in two branches, i.e. environmental samples (branch A) and fecal samples (branch B). The branch A composition was characterised by increased relative abundance of the *Aerococcu*s and *Corynebacterium* genera, similar to the results observed in the ordination analyses (**Figure 1C**). This branch was split into two sub-branches; i.e. sub-branch A1, dominated by species of the genera *Lactobacillus*, *Limosilactobacillus*, *Corynebacterium*, *Aerococcus* and *Staphylococcus*, and sub-branch A2, with *Aerococcus*, *Corynebacterium* and *Acinetobacter* as the main representatives, with lower *Lactobacillus* abundance. Sub-branch A1 included six out of the ten samples from WF and sub-branch A2 included mainly FD samples (six out of ten). Most of samples in branch B were fecal samples and further sub-clustering in this branch was influenced by the species present rather than differences in genera and reflected the time-point factor (**Figure 2**). Thus, the Feces 0dpw samples showed higher similarity with some Feces 7dpw samples (branch B2), and with Diarrhea 7dpw samples from ZnO-free group (branch B4). The remaining Feces 7dpw and Feces 14dpw samples were allocated to branch B3.

### 3.3 The intestinal microbiota of the piglet at weaning is different from the environmental microbiota and evolves towards anaerobic species’ dominance

**Figure 3:**
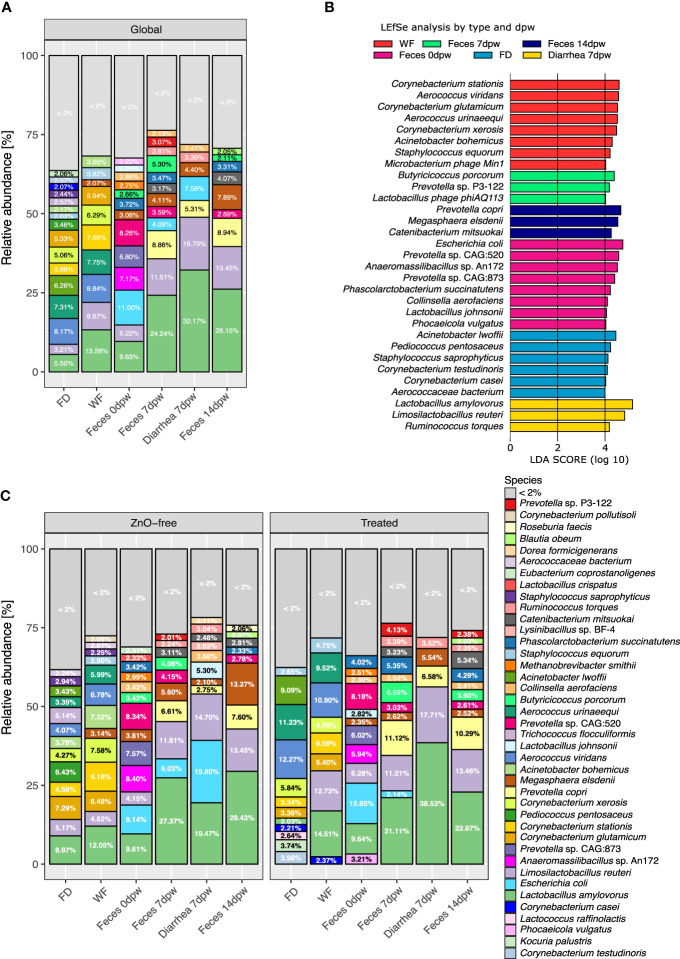
Microbiome composition in environmental and fecal samples from weaning pigs from 10 commercial farms. **(A)** Mean relative abundance of the most representative species in each type-dpw group. **(B)** Differences in species abundance, returned by LEfSe analysis, most likely explaining the differences among type and day post weaning variables. **(C)** Mean relative abundance of the main species in each type-dpw group splitted by treatment. * < 2%, refers to those species accounting for less than 2 percent of abundance. Taxonomic assignment was performed using Metaphlan3.

**Figure 3A** summarises the mean relative abundance of the most representative species in each sample type collected in the study. Environmental samples and Feces 0dpw showed similar or homogeneous abundance of different species while there was a hierarchy in microbiota relative abundance values in Feces 7dpw (including Diarrhea 7dpw) and Feces 14dpw samples. Focusing on fecal results, during the first 14 days after weaning, we observed an increase in abundance of the dominant species, shifts in species from the same genus and the bloom of anaerobes. *Lactobacillus amylovorus* and *Limosilactobacillus reuteri*, the two dominant species in fecal samples, increased in abundance (*P* < 0.05) during the post-weaning period (**Supplementary Table S3**). Feces 0dpw showed an even species composition, with the exclusive presence of *Prevotella* sp. CAG:873 and *Anaeromassilibacilus* sp. An172 (both with relative abundance values over 2%) and higher abundance of *Prevotella* sp. CAG:520 (**Figure 3B**). Other species associated with 0dpw feces were *Escherichia coli, Phascolarctobacterium succinatutens, Collinsella aerofaciens, Lactobacillus johnsonii* and *Phocaeicola vulgatus*. In subsequent samplings, i.e., 7dpw and 14dpw, the abundance of *Prevotella* sp. CAG:873 decreased, while the abundance of two other species of *Prevotella* increased, *Prevotella* sp. P3-122 at 7dpw and *Prevotella copri* at 14dpw (**Supplementary Table S3**). Two species exhibited higher abundance by linear discriminant analyses (LDA) at 7dpw; *Butyricicoccus porcorum* and the virus Lactobacillus phage phiAQ133. *Megasphaera elsdenii* increased in abundance towards the end of the of the study period with higher abundance at 14dpw (**Figure 3B**), sampling time at which we also observed higher abundance of *Catenibacterium mitsuokai*. While the relative abundance *M. elsdenii* in the piglets fecal microbiota was 3.08% at 0dpw, it had increased to 7.89% by 14dpw. In contrast, we observed the opposite trend for *E. coli*, from 11% at 0dpw to <2% at 14dpw. Diarrhea 7dpw samples showed a pattern which resembled those of 7dpw feces samples but with increased abundance of *E. coli*, and higher abundance of already dominant species such as *L. amylovorus*, *L. reuteri* and *Ruminococcus torques*. Both environmental samples showed similar compositions, with a high relative abundance of aerobic species such as *Aerococcus* spp. (*A. viridans, A. urinaeequi*) and *Corynebacterium spp* (*C. stationis, C. xerosis, C. glutamicum*).

### 3.4 In-feed antibiotic and zinc oxide impact taxonomic microbial composition both in environment and fecal samples

**Figure 4:**
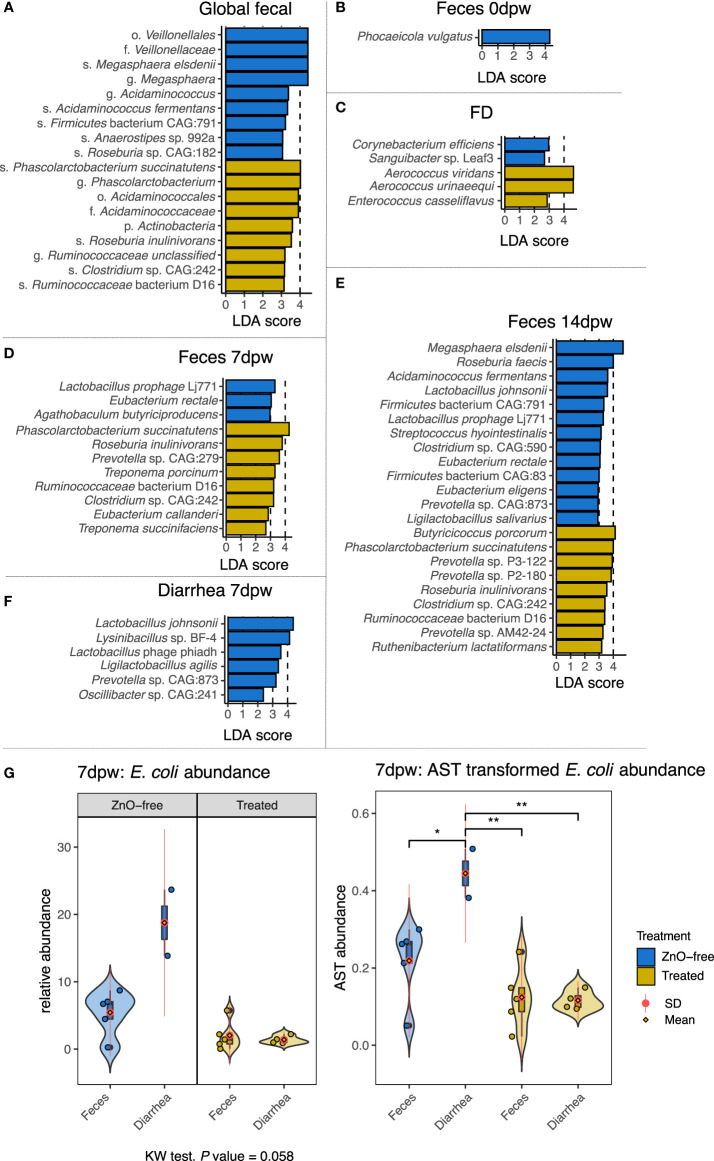
Differences in species abundance among dietary treatments in each sample type and day post weaning. Horizontal bars are coloured according to dietary treatment. **(A)** Global differences in species abundance by treatment. **(B)** Feces 0dpw. **(C)** FD. **(D)**. Feces 7dpw. **(E)** Feces 14dpw. **(F)** Diarrhea 7dpw. **(G)** Relative abundance and arcsine square root transformed abundance of *Escherichia coli* in samples of feces and diarrhea coloured by treatment. Points represent samples’ relative abundances values. Probability density curves at each values of abundance for each type of samples are depicted across each range of abundance values of each type of sample. Boxplots summarizing relative abundance values are included within the density curves. The lower, medium, and upper horizontal box lines correspond to the first, second and third quartiles (the 25th, 50th and 75th percentiles). Upper and lower whiskers include the range of the upper and lower points within the 1.5 interquartile range. The standard deviation (SD) and mean value of each type of sample are depicted as a red line and an orange diamond. *P < 0.05 and **P < 0.01, respectively. Taxonomic assignment was performed using Metaphlan3.

**Figure 3C** shows the comparison of the taxonomic profiles at species level between the samples from the Treated and ZnO-free farms for each sample type. The LEfSe LDA analyses of differential abundance run globally revealed different species associated with treatment (**Figure 4A**). Overall, dominance of *Lactobacillus* spp. was not affected by the treatment and global analysis of species abundance by LDA confirmed these differences among treatments for *M. elsdenii* and *P. succinatutens* abundance. Analysis by sample type and dpw variables (**Figures 4B–D**) showed that *P. vulgatus* was the only species found to be more abundant in feces from farms using in-feed antibiotics and ZnO at weaning, i.e. before the treatment began (**Figure 4B**). We observed a notable change in dominance of environmental species by treatment variable. Species associated with treatment in the FD microbiota were *A. viridans* and *A. urinaeequii* and *Enterococcus casseliflavus*, whereas *Sanguibacter* sp. Leaf3 and *Corynebacterium efficiens* were associated with FD samples from ZnO-free farms (**Figure 4C**).

The species *P. succinatutens*, *Roseburia inulinivorans*, *Ruminococcaceae* bacterium D16 and *Clostridium* sp. CAG:242 were also higher in abundance in feces from Treated farms both at 7dpw and 14dpw (**Figures 4D, E**), whereas different species of *Prevotella* spp. were associated with the fecal microbiota in Treated farms at different time points. In contrast, species more abundant in ZnO-free farms were: *Eubacterium rectale* along with the virus Lactobacillus phage Lj711 in both fecal samples at 7dpw and 14dpw (**Figures 4D, E**); *L. johnsonii* and *Prevotella* sp. CAG:873 in both Feces 14dpw and Diarrhea 7dpw (**Figures 4E, F**); and *Ligilactobacillus salivarius* and *Ligilactobacillus agilis* in Feces 14dpw and Diarrhea 7dpw samples, respectively. Other species enriched in feces from ZnO-free farms at 14dpw were *Eubacterium eligens*, *Clostridium* sp. CAG:590, *Streptococcus hyointestinalis*, *Acidaminococcus fermentans*, *Roseburia faecis* and *M. elsdenii.*

**Figure 5:**
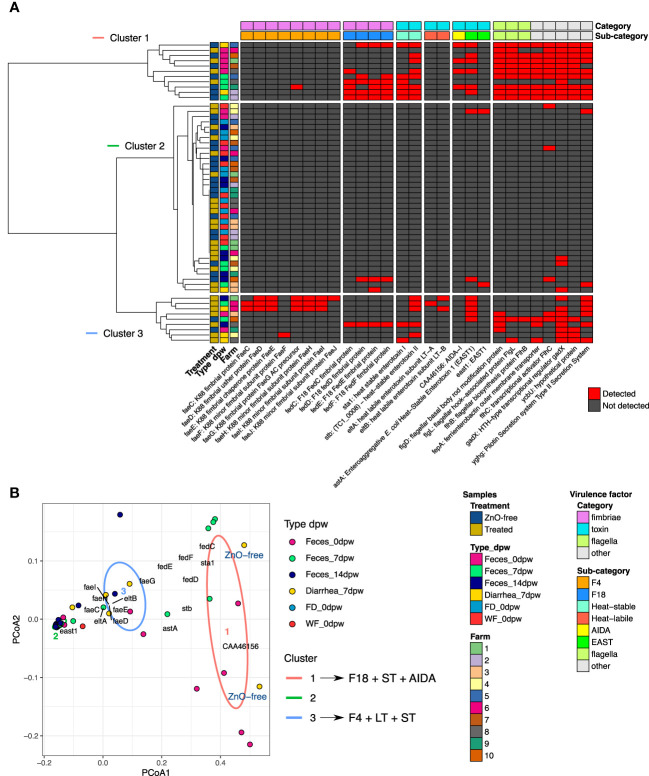
Summary of the *E. coli* virulence factors detected in assembled contigs of metagenomic sequencing reads. **(A)** heatmap of the virulence genes detected in each analysed sample and clustered by factors under study and by virulence category. **(B)** Principal coordinate analysis using Simple matching coefficient distance of samples by their virulence profile. Ellipses drawn on Figure **(B)** represent each Cluster group, with their shape being defined by the covariance within each group. Screening of contigs for *E. coli* virulence factors was performed using ABRicate.

Analysis of *E. coli* abundance revealed a trend and a high within-group variability (**Figure 4G**). Analysis of variance stabilizing AST transformed *E. coli* abundance revealed greater *E. coli* abundance in Diarrhea 7dpw samples from ZnO-free farms (**Figure 4G**). Further analyses of virulence factors linked to *E. coli* identified adhesins (*fimbriae* factors) and toxins associated to ETEC as shown in **Figure 5**. Enterotoxigenic *E. coli* associated genes were present in all diarrhea samples, regardless the treatment group. Hierarchical cluster analysis based on the virulence factor profile revealed three different clusters. Cluster 1 included ZnO-free Diarrhea 7dpw and 0dpw fecal samples, and was characterised by the presence of F18 fimbrial, heat-stable (ST) enterotoxin, and EAST1 toxin genes. Cluster 3 defined by F4 (K88), heat-labile (LT) and ST toxins genes, consisted of Treated Diarrhea 7dpw samples (**Figure 5A**). The ordination of virulence factor profiles grouped the samples according to the observed clusters, with cluster 2 composed of environmental samples and some 7 and 14dpw fecal samples (**Figure 5B**). Analysis of abundance of sequenced assigned to Pfam protein families revealed higher abundance of heat stable enterotoxin ST and heat-stable *E. coli* enterotoxin 1 in ZnO-free diarrhea samples (**Supplementary Figure S2**).

### 3.5 In-feed antibiotics and ZnO modify the microbiota species functional profile and diarrhea samples from ZnO-free farms show aerobic processes triggered by *E. coli*

**Figure 6:**
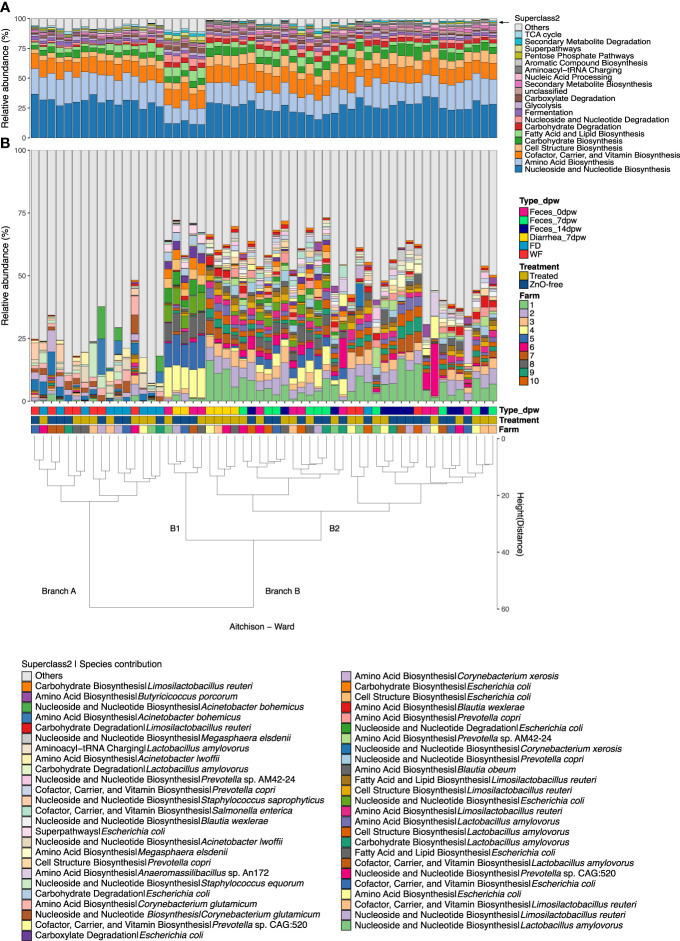
Relative abundance of the top 20 metacyc superclass2 grouped pathways. **(A)** Top 20 metacyc superclass2 grouped pathways. **(B)** Species contribution to each pathway (top 50 of pathways assumed by each species). Samples are clustered by their functional abundance profile using Ward clustering and the squared Aitchison distances between samples. Cluster dendrogram represents the similarity between samples regarding its functional and species composition. Variables information in each sample (from lower to upper level: Farm, Treatment, Type and day post weaning) are indicated in the coloured squares below the bars. Metabolic profiling was performed using HUMAnN3.

Analysis of the relative abundance of functional genes, grouped by superclass 2 level of MetaCyc metabolic pathways database, revealed the dominance of three functional superclasses, which accounted for more than 50% of the relative abundance of functional genes within the fecal microbiota: (i) nucleoside and nucleotide biosynthesis, (ii) amino acid biosynthesis, and iii) cofactor, carrier and vitamin biosynthesis (**Figure 6A**). The microbiota functional profiles, shown in **Figure 6**, are ordered according to Ward clustering using Aitchison distance. Most of the samples analysed were clustered by their environmental or fecal origin (branches A and B, respectively). We observed that in Feces 0dpw or ZnO-free Diarrhea 7dpw samples (**Figure 6B**, sub-branch B1), metabolic categories were ascribed to a lower number of species; whereas microbiota functions in Feces 7dpw and Feces 14dpw were built by a higher number of species (**Figure 6B**, sub-branch B2). Species contribution to metabolic categories varied across clusters, particularly in ZnO-free Diarrhea 7dpw samples, but also in species contribution of less dominant categories (**Figure 6B**).

**Supplementary Figure S3** shows which species significantly contributed to each superclass2 grouped metacyc pathway associated with the three factors under study, i.e., sample type, dpw and treatment. As already mentioned, the dominant functions were associated with different species to depending on the sample type, dpw and treatment. Notably, the Diarrhea 7dpw samples from ZnO-free farms were enriched in aerobic processes encoded by *E. coli*.

## 4 Discussion

Weaning in commercial pig farms is a critical time in the in the pig lifecycle. At weaning, the immune factors supplied by colostrum and milk are lost and the competitive exclusion exerted by the microbes resident in the intestine is weakened due to a new diet and stress. This often results in PWD which is traditionally managed using in-feed prophylactic antibiotics and therapeutic ZnO in many countries (Gresse et al., 2017). The recent ban on prophylactic AMU and therapeutic use of ZnO in the EU creates new challenges in the control of PWD. Some farmers ceased using these treatments before the change in regulations came into effect, providing an excellent opportunity to compare how their use or removal affects the intestinal microbiota after weaning. Studying the changes occurring in microbiota due to antibiotic and ZnO treatment and investigating potential associated biomarkers may open new opportunities to intervene during post-weaning period without antibiotic and/or ZnO use. In this study, we sequenced the environmental microbiota of clean weaning rooms and fecal microbiotas of piglets in the first two weeks post-weaning on farms still regularly using in-feed antibiotics and ZnO and compared it to farms that stopped using these treatments at least 3 years ago.

One of the goals of this study was to evaluate if the background microbiota of the rooms where piglets are allocated has any influence in the fecal microbiota of piglets. Both sample types collected, i.e., feeder-drinkers and wall-floor pen surfaces, exhibited high microbial richness and evenness, represented mainly by aerobic microorganisms. The environmental microbiota results were not different between farms using or not using antibiotics and ZnO. At the same time, environmental microbiota had only a weak influence on the fecal microbiota of piglets. Some previous studies indicated a potential impact of the environment on the new-born human and piglet microbiota (Merrifield et al., 2016; Chen et al., 2018; Law et al., 2021). Thus, there is a need for further research in this area. Based on our results, weaning (i.e. diet change from milk to feed and age) and in-feed treatments seem to have a larger impact than the environmental microbiota on the post weaning piglet fecal microbiota, although further studies are required.

Piglets at weaning (0dpw) exhibited a microbiome distinct from the 7dpw and 14dpw samples. This might be explained by a greater species and functional evenness, and the presence of some species in higher abundance than subsequent samplings e.g., *Prevotella* sp. CAG:873 and *Anaeromassilibacillus* sp. An172 in piglets at 0dpw. From weaning onwards, the microbiota composition shifted towards the dominance of *L. amylovorus*, *L. reuteri*, *P. copri* or *M. elsdenii*. A previous study has already demonstrated the intestinal colonisation of bacteria such as *P. copri* and *M. elsdenii* (Wang et al., 2019) in post-weaning. This temporal shift was clearly evidenced in the ordination plots which grouped fecal metagenomes on the basis of (i) Feces 0dpw, (ii) Feces 7dpw and 14dpw, and (iii) Diarrhea 7dpw. At the functional gene level, the differences were more subtle; functional profiles were enriched in the number of contributing species towards 7dpw and 14dpw time points. Interestingly, different species within the same genus were identified in the period of study, data which demonstrates a potential succession of species or strains phylogenetically related during the first two weeks post-weaning. Thus, we found different species of *Prevotella* spp. shifting across weaning period. Previous studies of pig metataxonomics using 16S rRNA sequencing have reported a microbial shift from a milk-oriented microbiome in piglets (composed mainly of *Lactobacillus* and *Bacteroides*), to a more complex carbohydrate adapted microbiota (dominated mainly by genus *Prevotella*) (Frese et al., 2015). One of the main advantages of shotgun metagenome sequencing is the ability to achieve species level resolution, enabling the exploration of species succession even in a short-term period of two weeks post-weaning. In this sense, Treated pigs were associated to more species of *Prevotella* species than ZnO-free pigs, with the exception of *Prevotella* sp. CAG:873. This last species of *Prevotella* was associated with the Feces 0dpw microbiota in the analysis by Type-dpw (**Figures 3A, C**). Interestingly, this species was also enriched in ZnO-free piglets’ microbiota in Diarrhea 7dpw and Feces 14dpw samples in the analysis by treatment (**Figures 4F, E**).

Diarrhea is the final disease outcome of the intestinal dysfunction at weaning. It may be the consequence of malabsorption, linked to atrophy of intestinal villi, or a secretory diarrhea when ETEC is present (Luppi, 2017). Here, we evaluated the composition in diarrheic feces collected at 7dpw. Microbiome compositional analyses revealed that Diarrhea 7dpw samples from ZnO-free farms resembled the composition of the microbiome at weaning (0dpw). On farms using antibiotics and ZnO, Diarrhea 7dpw samples were similar to non-diarrheic samples collected at 7dpw and 14dpw. Specifically looking at *E. coli* abundance in samples collected at 7dpw revealed a higher abundance of *E. coli* in samples from ZnO-free farms in both normal and diarrheic fecal samples (**Figure 4G**). Further analysis of virulence factors revealed the presence of adhesins and toxins related genes regardless the treatment. These virulence factors are required for *E. coli* pathogenic adverse effects. This result demonstrates that the abundance differences observed in diarrhea samples were not ascribed to differences in the presence or absence of ETEC pathotypes between treatment groups. This information, together with the microbial succession observed at weaning, suggests that antibiotic and ZnO use favors the early transition and maturation of the gut microbiota, even in animals with enteric problems. Interestingly, microbiota analysis also revealed different metabolic profiles in diarrhea samples from ZnO-free pigs, with greater representation of metabolic processes linked to *E. coli*. Species associated with antibiotic and ZnO treatment within each type-dpw microbiota included representatives of the orders *Eubacteriales*, and *Bacteroidales* (Prevotella spp), and, to a lesser extent to *Spirochaetales* and *Acidaminococcales*. *P. succinatutens*, belonging to the *Acidaminococcales* order, was a common species enriched in Treated animals both at 7 and 14dpw. This species is a succinate utilizing anaerobic bacteria that has been reported to increase in abundance because of antibiotic treatment (Yan et al., 2020). Similarly, based on results in the current and previous studies (Soler et al., 2018; Ghanbari et al., 2019; Ortiz Sanjuán et al., 2022), *Prevotella* seems to be a genus within which many representatives are resistant to ZnO, which becomes favored by antimicrobial treatments.

The microbiota from ZnO-free farms were enriched with *Lactobacillus* spp. and members of the class *Bacilli*. The dominance of *L. amylovorus* and *L. reuteri* observed across the sampling time points was not affected by treatment, whereas other lactobacilli e.g., *L. johnsonii*, *L. salivarius* and *L. agilis*, were affected by antibiotic and ZnO treatment. *L. amylovorus* and *L. reuteri* are two of the most common species from *Lactobacillaceae* family dominating pig’s intestinal microbiota (Højberg et al., 2005; Vahjen et al., 2010; Starke et al., 2014; Ortiz Sanjuán et al., 2022). In line with our previous study, these two species were not affected by ZnO (Ortiz Sanjuán et al., 2022). Regarding the other species of lactobacilli, ZnO has been previously reported to affect lactobacilli species in a different manner, and thus the susceptibility to ZnO might be species specific (Starke et al., 2014). *Eubacterium* is a genus found to be an important member of the human core microbiome, described as a butyrate-producing bacteria (Mukherjee et al., 2020). In this study, we found that two of the major species of interest within this genus (Mukherjee et al., 2020), *E. rectale* and *E. eligens*, were present at higher relative abundance on the ZnO-free farms. Another two species linked to ZnO-free herds were *M. elsdenii* and *Acidaminococcus fermentans*. Previous studies have also reported the negative impact of ZnO on the abundance of these two species (Pieper et al., 2020; Silva et al., 2021; Ortiz Sanjuán et al., 2022), which are capable of produce butyrate from lactate and glutamate, respectively (Tsukahara et al., 2006; Sarmikasoglou and Faciola, 2022; Böck, 2009). Both amino-acid fermenting species are common members of the adult pig fecal microbiota and increase in the abundance as the animal grows. In addition, *M. elsdenii* can use complex plant carbohydrates and produce short chain fatty acids (Shetty et al., 2013; Wang et al., 2019; Li et al., 2020). Our results demonstrate that antibiotics and ZnO limit and favor the growth of microorganisms with redundant functions but differential capacity to adapt to these two antimicrobial exposures. Considering the characteristics of the microorganisms mentioned, the taxonomic variations observed likely reflect the ability of certain microorganisms to colonise or outcompete bacteria with similar broad roles in the presence of antibiotics and ZnO and *vice versa*, that is, to adapt to the conditions generated by antibiotics and ZnO in the intestine, and perform the same broad metabolic functions, i.e. functional redundancy (**Figure 6A**) (Tian et al., 2020). These taxonomic changes were reflected as well in the metabolic activity of the microbiotas, which were associated with some of the aforementioned species. The most remarkable differences between treatments were found in the Feces 14dpw microbiota, with *M. elsdenii* associated with functional pathways on ZnO-free farms, whereas the microbiota functional pathways in Diarrhea 7dpw samples on ZnO-free farms were dominated by functions linked to *E. coli* with higher abundance of aerobic-related pathways such as the tricarboxylic acid (TCA) cycle or glycolysis. Impairment of strict anaerobic population and thriving of aerotolerant species as a consequence of intestinal inflammation has already been described as a strategy of some opportunistic enteric pathogens, such as *Salmonella enterica* or *E. coli* (Baümler and Sperandio, 2016; Gresse et al., 2017). The aerobic environment generated by the immune response provides an advantage to these facultative anaerobes, such as *E. coli*, in the microbiota from diarrheic ZnO-free samples.

In this study we show that the transition and maturation of the microbiota early after weaning is remarkably consistent among farms and is influenced more by dietary change post weaing, age, and in-feed medication than by the environmental microbiota. The use of antibiotics and ZnO altered the taxonomy and functionality of the fecal microbiota whereby bacteria such as *Prevotella* spp., *P. succinatutens*, *M. elsdenii* and, to a lesser extent, *E. coli*, colonised the intestinal niche in response to this treatment. Using next generation sequencing, we detected *E. coli* virulence factor related genes in all fecal samples. These results show that these treatments were efficient in controlling *E. coli* overgrowth at weaning, regardless of the presence or absence of these virulence factors. Results of this study show a snapshot of the fecal microbiota in pigs within the first two weeks after weaning, which can be useful to monitor taxonomic, functional composition, or even pathogen detection on pig herds (Munk et al., 2017). Further studies are necessary to evaluate the effects of antibiotics and ZnO on specific intestinal locations, at this level of description. The compositional and functional analysis of feces with diarrhea showed that antibiotic and ZnO treatment favored the microbial transition observed in healthy animals. These results demonstrate the microbial modulation, in taxa and functionality, and provide a reference in post-weaning microbiota to study potential strategies to replace antibiotics and ZnO.

## Data availability statement

The datasets presented in this study can be found in online repositories. The names of the repository/repositories and accession number(s) can be found below: https://www.ncbi.nlm.nih.gov/bioproject/?term=PRJNA894343, PRJNA894343.

## Ethics statement

The animal studies were approved by Teagasc Animal Ethics Committee. The studies were conducted in accordance with the local legislation and institutional requirements. Written informed consent was obtained from the owners for the participation of their animals in this study.

## Author contributions

JO: Conceptualization, Data curation, Formal analysis, Investigation, Methodology, Software, Visualization, Writing – original draft, Writing – review & editing. EM: Conceptualization, Funding acquisition, Investigation, Project administration, Resources, Supervision, Validation, Visualization, Writing – review & editing. RC-R: Formal analysis, Software, Supervision, Validation, Visualization, Writing – review & editing. FC: Methodology, Resources, Supervision, Validation, Writing – review & editing. PC: Methodology, Resources, Supervision, Validation, Writing – review & editing. JG: Conceptualization, Methodology, Supervision, Validation, Writing – review & editing. DE: Conceptualization, Data curation, Formal analysis, Methodology, Writing – review & editing. HA: Conceptualization, Investigation, Supervision, Validation, Visualization, Writing – review & editing. LO'N: Conceptualization, Writing – review & editing, Validation.

## References

[B1] Baümler A. J. Sperandio V. (2016). Interactions between the microbiota and pathogenic bacteria in the gut. Nature 535 (7610), 85–93. doi: 10.1038/nature18849 27383983 PMC5114849

[B2] Beghini F. McIver L. J. Blanco-Míguez A. Dubois L. Asnicar F. Maharjan S.. (2021). Integrating taxonomic, functional, and strain-level profiling of diverse microbial communities with biobakery 3. ELife 10, 1–42. doi: 10.7554/eLife.65088 PMC8096432 33944776

[B3] Benjamini Y. Hochberg Y. (1995). Controlling the false discovery rate: A practical and powerful approach to multiple testing. J. R. Stat. Society. Ser. B (Methodological) 5 (1), 289–3005. doi: 10.1111/j.2517-6161.1995.tb02031.x

[B4] Bolger A. M. Lohse M. Usadel B. (2014). Trimmomatic: A flexible trimmer for illumina sequence data. Bioinformatics 30 (15), 2114–2205. doi: 10.1093/bioinformatics/btu170 24695404 PMC4103590

[B5] Böck A. (2009). “Fermentation”. In Encyclopedia of Microbiology (Third Edition) edited byMoselio S. (Cambridge, MA, USA: Academic Press), 2114–2205.

[B6] Bonetti A. Tugnoli B. Piva A. Grilli E. (2021). Towards zero zinc oxide: feeding strategies to manage post-weaning diarrhea in piglets. Animals 11 (3), 1–24. doi: 10.3390/ani11030642 PMC7997240 33670980

[B7] Broom L. J. Miller H. M. Kerr K. G. Knapp J. S. (2006). Effects of zinc oxide and enterococcus faecium SF68 dietary supplementation on the performance, intestinal microbiota and immune status of weaned piglets. Res. Veterinary Sci. 80 (1), 45–545. doi: 10.1016/j.rvsc.2005.04.004 15946717

[B8] Bushnell B. (2014). BBMap: A Fast, Accurate, Splice-Aware Aligner (Berkeley, CA (United States). Available at: https://www.osti.gov/servlets/purl/1241166.

[B9] Caspi R. Billington R. Keseler I. M. Kothari A. Krummenacker M. Midford P. E.. (2020). The metaCyc database of metabolic pathways and enzymes - a 2019 update. Nucleic Acids Res. 48 (D1), D445–D453. doi: 10.1093/nar/gkz862 31586394 PMC6943030

[B10] Chen X. Xu J. Ren E. Su Y. Zhu W. (2018). Co-occurrence of early gut colonization in neonatal piglets with microbiota in the maternal and surrounding delivery environments. Anaerobe 49, 30–40. doi: 10.1016/j.anaerobe.2017.12.002 29223548

[B11] Dou S. Gadonna-Widehem P. Rome V. Hamoudi D. Rhazi L. Lakhal L.. (2017). Characterisation of early-life fecal microbiota in susceptible and healthy pigs to post-weaning diarrhoea. PloS One 12 (1), e0169851. doi: 10.1371/journal.pone.0169851 28072880 PMC5225014

[B12] European Commission (2019). Regulation (EU) 2019/6 of the European parliament and of the council of 11 December 2018 on veterinary medicinal products and repealing directive 2001/82/EC. Off. J. Eur. Union L4 (726), 43–167. Available at: https://eur-lex.europa.eu/legal-content/EN/TXT/PDF/?uri=CELEX:32019R0006.

[B13] European Commission (2019). Regulation (EU) 2019/4 of the European parliament and of the council of 11 December 2018 on the manufacture, placing on the market and use of medicated feed, amending regulation (EC) no 183/2005 of the European parliament. Oj L4 (7.1.2019), 1–23. Available at: https://eur-lex.europa.eu/legal-content/EN/TXT/PDF/?uri=CELEX:32019R0004&from=EN.

[B14] Fairbrother J. M. Nadeau É. (2019). “Colibacillosis,” in Diseases of Swine. Eds. Zimmerman J. J. Karriker L. A. Ramirez A. Schwartz K. J. Stevenson G. W. Zhang J. Eleventh E. (Hoboken, New Jersey, USA: Wiley), 807–834. doi: 10.1002/9781119350927.ch52

[B15] Finn R. D. Mistry J. Tate J. Coggill P. Heger A. Pollington J. E.. (2010). The pfam protein families database. Nucleic Acids Res. 38 (suppl_1), D211–D222. doi: 10.1093/nar/gkp985 19920124 PMC2808889

[B16] Frese S. A. Parker K. Chris Calvert C. Mills D. A. (2015). Diet shapes the gut microbiome of pigs during nursing and weaning. Microbiome 3 (1), 1–105. doi: 10.1186/s40168-015-0091-8 26167280 PMC4499176

[B17] Ghanbari M. Klose V. Crispie F. Cotter P. D. (2019). The dynamics of the antibiotic resistome in the feces of freshly weaned pigs following therapeutic administration of oxytetracycline. Sci. Rep. 9 (1), 1–115. doi: 10.1038/s41598-019-40496-8 30858509 PMC6411716

[B18] Graves S. Piepho H.-P. Selzer L. Dorai-Raj S. (2019). multcompView: Visualizations of Paired Comparisons.

[B19] Gresse R. Chaucheyras-Durand F. Fleury M. A. Wiele T. V. d. Forano E. Blanquet-Diot S. (2017). Gut microbiota dysbiosis in postweaning piglets: understanding the keys to health. Trends Microbiol. 25 (10), 851–735. doi: 10.1016/j.tim.2017.05.004 28602521

[B20] Højberg O. Canibe N. Poulsen H. D. Hedemann M. S. Jensen. B. B. (2005). Influence of dietary zinc oxide and copper sulfate on the gastrointestinal ecosystem in newly weaned piglets. Appl. Environ. Microbiol. 71 (5), 2267–2277. doi: 10.1128/AEM.71.5.2267-2277.2005 15870311 PMC1087531

[B21] Inkscape Project (2020) Inkscape. Available at: https://inkscape.org.

[B22] Jari Oksanen F. Blanchet G. Friendly M. Kindt R. Legendre P. McGlinn D.. (2020) Vegan: Community Ecology Package. Available at: https://cran.r-project.org/package=vegan.

[B23] Langmead B. Salzberg S. L. (2012). Fast gapped-read alignment with bowtie 2. Nat. Methods 9 (4), 357–595. doi: 10.1038/nmeth.1923 22388286 PMC3322381

[B24] Law K. Lozinski B. Torres I. Davison S. Hilbrands A. Nelson E.. (2021). Disinfection of maternal environments is associated with piglet microbiome composition from birth to weaning. MSphere 6 (5), 1–175. doi: 10.1128/msphere.00663-21 PMC8550216 34494881

[B25] Lenth R. V. (2016). Least-squares means: the R package lsmeans. J. Stat. Software 69 (1). doi: 10.18637/jss.v069.i01

[B26] Li Y. Zhu Y. Wei H. Chen Y. Shang H. (2020). Study on the diversity and function of gut microbiota in pigs following long-term antibiotic and antibiotic-free breeding. Curr. Microbiol. 77 (12), 4114–4285. doi: 10.1007/s00284-020-02240-8 33067706

[B27] Liu C. Cui Y. Li X. Yao M. (2021). Microeco: an R package for data mining in microbial community ecology. FEMS Microbiol. Ecol. 97 (2). doi: 10.1093/femsec/fiaa255 33332530

[B28] Luppi A. (2017). Swine enteric colibacillosis: diagnosis, therapy and antimicrobial resistance. Porcine Health Manage. 3, 1–18. doi: 10.1186/s40813-017-0063-4 PMC5547460 28794894

[B29] Martinez Arbizu P. (2020). PairwiseAdonis: pairwise multilevel comparison using adonis. Available at: https://github.com/pmartinezarbizu/pairwiseAdonis.

[B30] Merrifield C. A. Lewis M. C. Berger B. Cloarec O. Heinzmann S. S. Charton F.. (2016). Neonatal environment exerts a sustained influence on the development of the intestinal microbiota and metabolic phenotype. ISME J. 10 (1), 145–157. doi: 10.1038/ismej.2015.90 26066712 PMC4681865

[B31] Mukherjee A. Lordan C. Paul Ross R. Cotter P. D. (2020). Gut microbes from the phylogenetically diverse genus eubacterium and their various contributions to gut health. Gut Microbes 12 (1), 1–285. doi: 10.1080/19490976.2020.1802866 PMC7524325 32835590

[B32] Munk P. Andersen V. D. Knegt L. D. Jensen M. S. Knudsen B. E. Lukjancenko O.. (2017). A sampling and metagenomic sequencing-based methodology for monitoring antimicrobial resistance in swine herds. J. Antimicrobial Chemotherapy 72 (2), 385–392. doi: 10.1093/jac/dkw415 28115502

[B33] Nurk S. Meleshko D. Korobeynikov A. Pevzner P. A. (2017). MetaSPAdes: A new versatile metagenomic assembler. Genome Res. 27 (5), 824–345. doi: 10.1101/gr.213959.116 28298430 PMC5411777

[B34] Ortiz Sanjuán J. M. Manzanilla E. G. Cabrera-Rubio R. Crispie F. Cotter P. D. Garrido J. J.. (2022). Using shotgun sequencing to describe the changes induced by in-feed zinc oxide and apramycin in the microbiomes of pigs one week postweaning. Microbiol. Spectr. 10 (4). doi: 10.1128/spectrum.01597-22 PMC9431492 35950862

[B35] Pasquet J. Chevalier Y. Pelletier J. Couval E. Bouvier D. Bolzinger M. A. (2014). The contribution of zinc ions to the antimicrobial activity of zinc oxide. Colloids Surfaces A: Physicochemical Eng. Aspects 457 (1), 263–745. doi: 10.1016/j.colsurfa.2014.05.057

[B36] Pieper R. Dadi T. H. Pieper L. Vahjen W. Franke A. Reinert K.. (2020). Concentration and chemical form of dietary zinc shape the porcine colon microbiome, its functional capacity and antibiotic resistance gene repertoire. ISME J. 14 (11), 2783–2935. doi: 10.1038/s41396-020-0730-3 32747713 PMC7784847

[B37] Poulsen H. D. (1995). Zinc oxide for weanling piglets. Acta Agriculturae Scandinavica A: Anim. Sci. 45 (3), 159–167. doi: 10.1080/09064709509415847

[B38] Raivo K. (2019) Pheatmap: Pretty Heatmaps. Available at: https://cran.r-project.org/package=pheatmap.

[B39] Rhouma M. Fairbrother J. M. Beaudry F. Letellier A. (2017). Post weaning diarrhea in pigs: risk factors and non-colistin-based control strategies. Acta Veterinaria Scandinavica 59 (1), 1–195. doi: 10.1186/s13028-017-0299-7 28526080 PMC5437690

[B40] Sales J. (2013). Effects of pharmacological concentrations of dietary zinc oxide on growth of post-weaning pigs: A meta-analysis. Biol. Trace Element Res. 152 (3), 343–349. doi: 10.1007/s12011-013-9638-3 23463368

[B41] Sarmikasoglou E. Faciola A. P. (2022). Ruminal bacteria lipopolysaccharides: an immunological and microbial outlook. J. Anim. Sci. Biotechnol. 13 (1), 1–7. doi: 10.1186/s40104-022-00692-5 35418112 PMC9008999

[B42] Segata N. Izard J. Waldron L. Gevers D. Miropolsky L. Garrett W. S.. (2011). Metagenomic biomarker discovery and explanation. Genome Biol. 12 (6), R60. doi: 10.1186/gb-2011-12-6-r60 21702898 PMC3218848

[B43] Shetty S. A. Marathe N. P. Lanjekar V. Ranade D. Shouche Y. S. (2013). Comparative genome analysis of megasphaera sp. Reveals niche specialization and its potential role in the human gut. PloS One 8 (11), e79353. doi: 10.1371/journal.pone.0079353 24260205 PMC3832451

[B44] Silva C. A. d. Bentin L. A. T. Dias C. P. Callegari M. A. Facina V. B. Dias F. T. F.. (2021). Impact of zinc oxide, benzoic acid and probiotics on the performance and cecal microbiota of piglets. Anim. Microbiome 3 (1), 86. doi: 10.1186/s42523-021-00151-y 34930490 PMC8686666

[B45] Sloup V. Jankovská I. Nechybová S. Peřinková P. Langrová I. (2017). Zinc in the animal organism: A review. Scientia Agriculturae Bohemica 48 (1), 13–215. doi: 10.1515/sab-2017-0003

[B46] Söderberg T. A. Sunzel B. Holm S. Elmros T. Hallmans G. Sjöberg S. (1990). Antibacterial effect of zinc oxide in vitro. Scandinavian J. Plast. Reconstructive Surg. Handb. Surg. 24 (3), 193–975. doi: 10.3109/02844319009041278 2281305

[B47] Sokal R. R. Michener C. D. (1958). A statistical methods for evaluating relationships. Univ. Kansas Sci. Bull. 38, 1409–1448. Available at: https://ia800703.us.archive.org/5/items/cbarchive_33927_astatisticalmethodforevaluatin1902/astatisticalmethodforevaluatin1902.pdf.

[B48] Soler C. Goossens T. Bermejo A. Migura-garcı L. Cusco A. Francino O.. (2018). Digestive Microbiota Is Different in Pigs Receiving Antimicrobials or a Feed Additive during the Nursery Period . PLoS ONE Zoetendal E. G. (NETHERLANDS: Wageningen Universiteit) 13 (5), e0197353. doi: 10.1371/journal.pone.0197353 PMC5969774 29799833

[B49] Spees A. M. Wangdi T. Lopez C. A. Kingsbury D. D. Xavier M. N. Winter S. E.. (2013). Streptomycin-induced inflammation enhances escherichia coli gut colonization through nitrate respiration. MBio 4 (4), 1–105. doi: 10.1128/mBio.00430-13 PMC3705454 23820397

[B50] Standing Committee on veterinary medicinal products (2017) COMMISSION IMPLEMENTING DECISION of 26.6.2017 Concerning the Marketing Authorisations for Veterinary Medicinal Products Containing ‘Zinc Oxide’ to Be Administered Orally to Food Producing Species. Available at: https://ec.europa.eu/health/documents/community-register/2017/20170626136754/dec_136754_en.pdf.

[B51] Starke I. C. Pieper R. Neumann K. Zentek J. Vahjen W. (2014). The impact of high dietary zinc oxide on the development of the intestinal microbiota in weaned piglets. FEMS Microbiol. Ecol. 87 (2), 416–427. doi: 10.1111/1574-6941.12233 24118028

[B52] Team, R Core (2022). R: A Language and Environment for Statistical Computing Vol. 4 (Vienna, Austria: R Foundation for Statistical Computing), 15.

[B53] Tian L. Wang X.-W. Wu A.-K. Fan Y. Friedman J. Dahlin A.. (2020). Deciphering functional redundancy in the human microbiome. Nat. Commun. 11 (1), 6217. doi: 10.1038/s41467-020-19940-1 33277504 PMC7719190

[B54] Tsukahara T. Hashizume K. Koyama H. Ushida K. (2006). Stimulation of Butyrate Production through the Metabolic Interaction among Lactic Acid Bacteria, Lactobacillus Acidophilus, and Lactic Acid-Utilizing Bacteria, Megasphaera Elsdenii, in Porcine Cecal Digesta. Anim. Sci. J. 77 (4), 454–461. doi: 10.1111/j.1740-0929.2006.00372.x

[B55] Vahjen W. Pieper R. Zentek J. (2010). Bar-coded pyrosequencing of 16S rRNA gene amplicons reveals changes in ileal porcine bacterial communities due to high dietary zinc intake. Appl. Environ. Microbiol. 76 (19), 6689–6691. doi: 10.1128/AEM.03075-09 20709843 PMC2950476

[B56] Wang X. Tsai T. Deng F. Wei X. Chai J. Knapp J.. (2019). Longitudinal investigation of the swine gut microbiome from birth to market reveals stage and growth performance associated bacteria. Microbiome 7 (1), 1–18. doi: 10.1186/s40168-019-0721-7 31362781 PMC6664762

[B57] Wątły J. Potocki S. Rowińska-Żyrek M. (2016). Zinc homeostasis at the bacteria/host interface—From coordination chemistry to nutritional immunity. Chem. - A Eur. J. 22 (45), 15992–15105. doi: 10.1002/chem.201602376 27555527

[B58] Wickham H. (2016). Ggplot2: Elegant Graphics for Data Analysis (New York: Springer-Verlag). Available at: https://ggplot2.tidyverse.org.

[B59] Yan H. Yu B. Degroote J. Spranghers T. Van Noten N. Majdeddin M.. (2020). Antibiotic affects the gut microbiota composition and expression of genes related to lipid metabolism and myofiber types in skeletal muscle of piglets. BMC Veterinary Res. 16 (1), 1–12. doi: 10.1186/s12917-020-02592-0 PMC7568366 33066774

