# Supplementary material for: Fine-tuning of post-weaning pig microbiome structure and functionality by in-feed zinc oxide and antibiotics use
Source: Front Cell Infect Microbiol. 2024 Feb 7;14:1354449. doi: 10.3389/fcimb.2024.1354449 (PMC10879578; doi:10.3389/fcimb.2024.1354449)
Supplement: Supplementary file 1 [file Presentation_1.pdf]

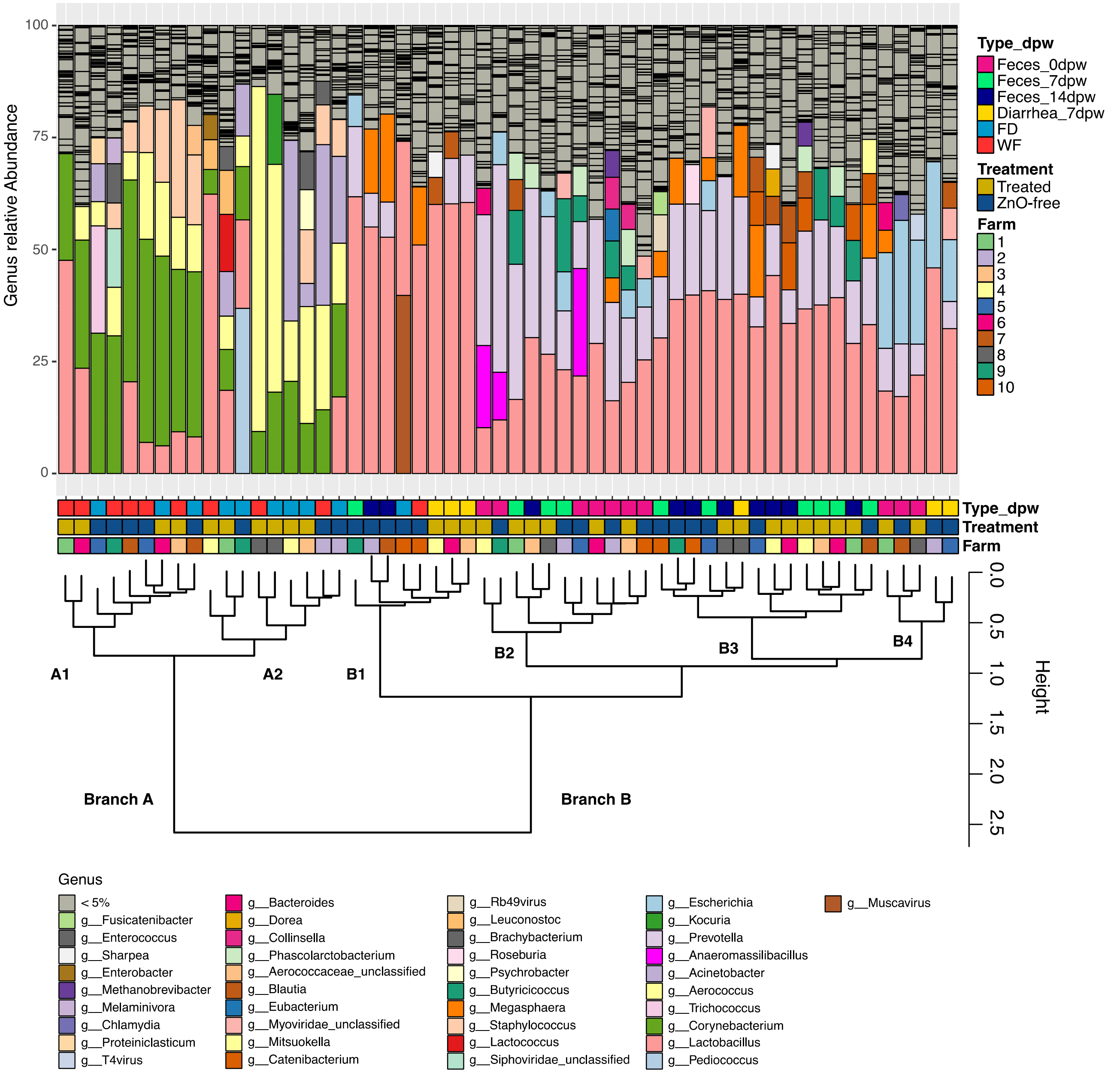

Supplementary Figure S1. Stacked bar plot of the relative abundance of the main genera in each sample analysed from 10 commercial farms. Profiles of samples are ordered by Ward clustering of the squared Weighted Unifrac distances between samples. Cluster dendrogram represents the similarity between samples regarding its microbial composition. Variables information in each sample (from lower to upper level: Farm, Treatment, Type-dpw) are indicated in the coloured squares below the bars. Taxonomic assignment was performed using Metaphlan3.
