# Supplementary material for: Fine-tuning of post-weaning pig microbiome structure and functionality by in-feed zinc oxide and antibiotics use
Source: Front Cell Infect Microbiol. 2024 Feb 7;14:1354449. doi: 10.3389/fcimb.2024.1354449 (PMC10879578; doi:10.3389/fcimb.2024.1354449)
Supplement: Supplementary file 2 [file Presentation_2.pdf]

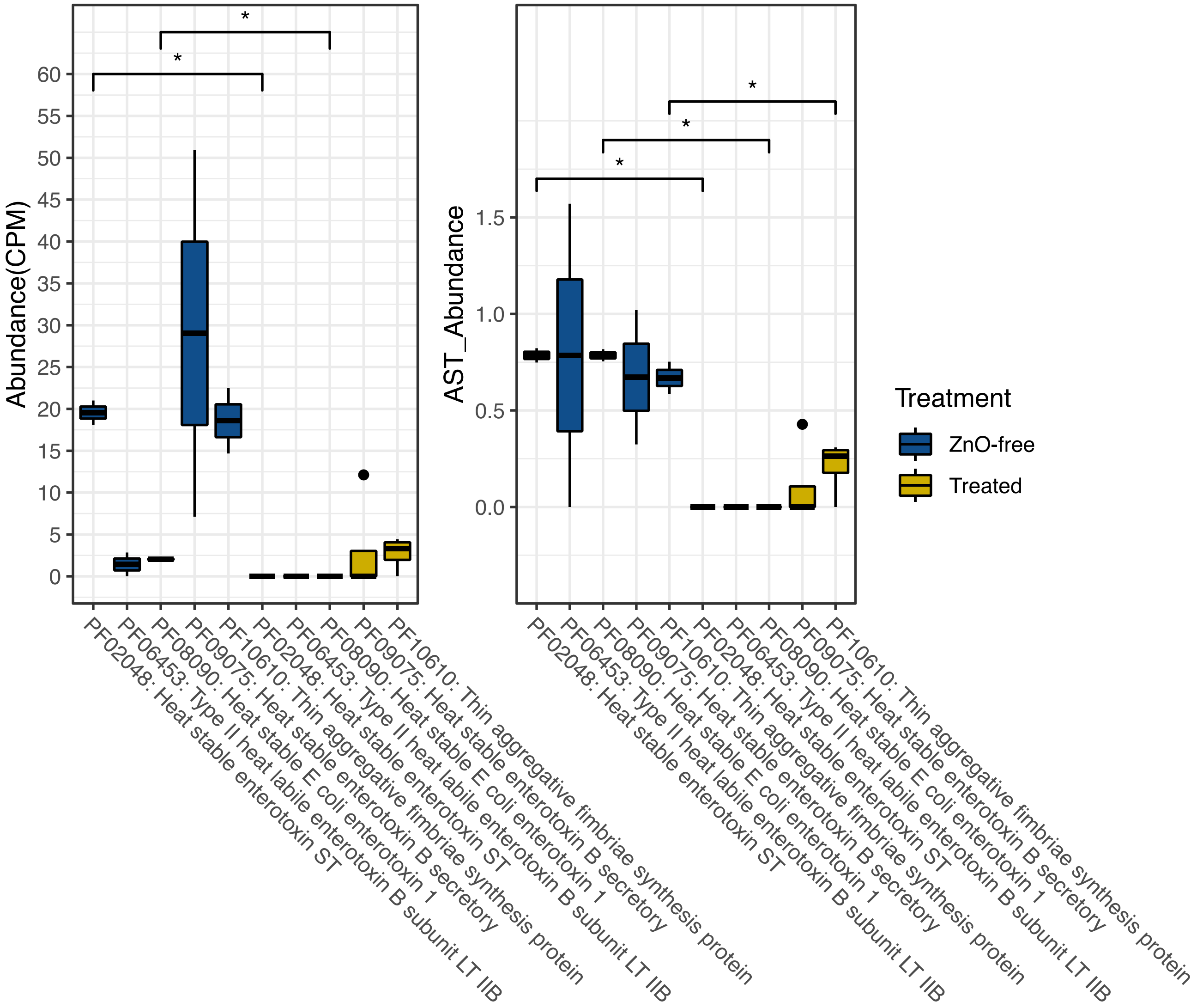

Supplementary Figure S2. Boxplots summarizing the abundance of Heat-Stable and Heat-Labile Pfam protein families in diarrhea samples, expressed in Counts per Million. The differences in the protein families abundances were evaluated using Kruskal-Wallis test. The lower, medium, and upper horizontal box lines correspond to the first, second and third quartiles (the 25th, 50th and 75th percentiles). Upper and lower whiskers include the range of the upper and lower points within the 1.5 interquartile range. *P < 0.05. Functional profiling of sequences was performed using HUMAnN3, and regrouped into PFAM protein domains.
