# Supplementary material for: Fine-tuning of post-weaning pig microbiome structure and functionality by in-feed zinc oxide and antibiotics use
Source: Front Cell Infect Microbiol. 2024 Feb 7;14:1354449. doi: 10.3389/fcimb.2024.1354449 (PMC10879578; doi:10.3389/fcimb.2024.1354449)
Supplement: Supplementary file 3 [file Presentation_3.pdf]

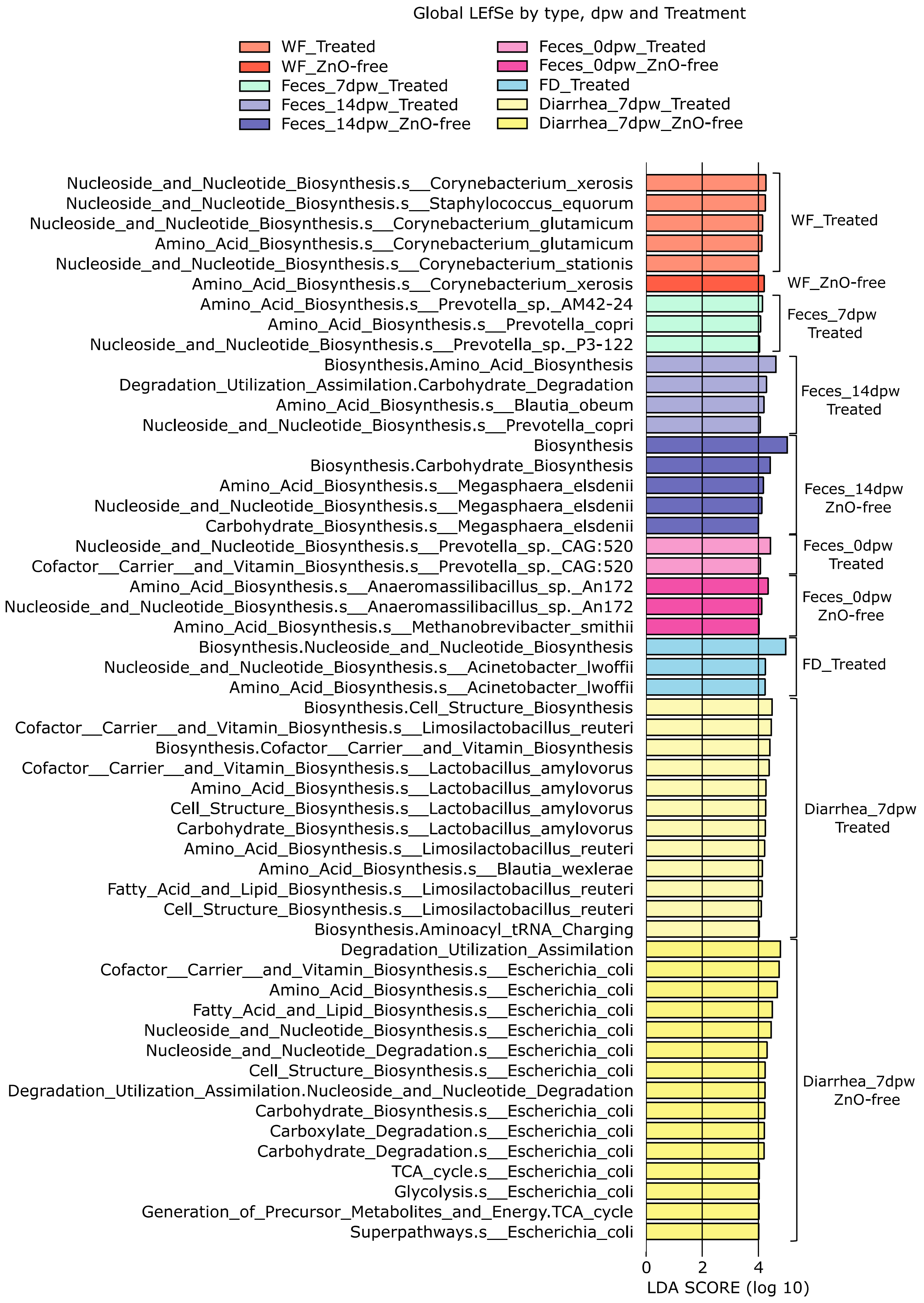

Global LEfSe by type, dpw and Treatment
WF_Treated
WF_ZnO-free
Feces_7dpw_Treated
Feces_14dpw_Treated
Feces_14dpw_ZnO-free
Feces_0dpw_Treated
Feces_0dpw_ZnO-free
FD_Treated
Diarrhea_7dpw_Treated
Diarrhea_7dpw_ZnO-free
Nucleoside_and_Nucleotide_Biosynthesis.s__Corynebacterium_xerosis
Nucleoside_and_Nucleotide_Biosynthesis.s__Staphylococcus_equorum
Nucleoside_and_Nucleotide_Biosynthesis.s__Corynebacterium_glutamicum
Amino_Acid_Biosynthesis.s__Corynebacterium_glutamicum
Nucleoside_and_Nucleotide_Biosynthesis.s__Corynebacterium_stationis
Amino_Acid_Biosynthesis.s__Corynebacterium_xerosis
Amino_Acid_Biosynthesis.s__Prevotella_sp._AM42-24
Amino_Acid_Biosynthesis.s__Prevotella_copri
Nucleoside_and_Nucleotide_Biosynthesis.s__Prevotella_sp._P3-122
Biosynthesis.Amino_Acid_Biosynthesis
Degradation_Utilization_Assimilation.Carbohydrate_Degradation
Amino_Acid_Biosynthesis.s__Blautia_obeum
Nucleoside_and_Nucleotide_Biosynthesis.s__Prevotella_copri
Biosynthesis
Biosynthesis.Carbohydrate_Biosynthesis
Amino_Acid_Biosynthesis.s__Megasphaera_elsdenii
Nucleoside_and_Nucleotide_Biosynthesis.s__Megasphaera_elsdenii
Carbohydrate_Biosynthesis.s__Megasphaera_elsdenii
Nucleoside_and_Nucleotide_Biosynthesis.s__Prevotella_sp._CAG:520
Cofactor__Carrier__and_Vitamin_Biosynthesis.s__Prevotella_sp._CAG:520
Amino_Acid_Biosynthesis.s__Anaeromassilibacillus_sp._An172
Nucleoside_and_Nucleotide_Biosynthesis.s__Anaeromassilibacillus_sp._An172
Amino_Acid_Biosynthesis.s__Methanobrevibacter_smithii
Biosynthesis.Nucleoside_and_Nucleotide_Biosynthesis
Nucleoside_and_Nucleotide_Biosynthesis.s__Acinetobacter_lwoffii
Amino_Acid_Biosynthesis.s__Acinetobacter_lwoffii
Biosynthesis.Cell_Structure_Biosynthesis
Cofactor__Carrier__and_Vitamin_Biosynthesis.s__Limosilactobacillus_reuteri
Biosynthesis.Cofactor__Carrier__and_Vitamin_Biosynthesis
Cofactor__Carrier__and_Vitamin_Biosynthesis.s__Lactobacillus_amylovorus
Amino_Acid_Biosynthesis.s__Lactobacillus_amylovorus
Cell_Structure_Biosynthesis.s__Lactobacillus_amylovorus
Carbohydrate_Biosynthesis.s__Lactobacillus_amylovorus
Amino_Acid_Biosynthesis.s__Limosilactobacillus_reuteri
Amino_Acid_Biosynthesis.s__Blautia_wexlerae
Fatty_Acid_and_Lipid_Biosynthesis.s__Limosilactobacillus_reuteri
Cell_Structure_Biosynthesis.s__Limosilactobacillus_reuteri
Biosynthesis.Aminoacyl_tRNA_Charging
Degradation_Utilization_Assimilation
Cofactor__Carrier__and_Vitamin_Biosynthesis.s__Escherichia_coli
Amino_Acid_Biosynthesis.s__Escherichia_coli
Fatty_Acid_and_Lipid_Biosynthesis.s__Escherichia_coli
Nucleoside_and_Nucleotide_Biosynthesis.s__Escherichia_coli
Nucleoside_and_Nucleotide_Degradation.s__Escherichia_coli
Cell_Structure_Biosynthesis.s__Escherichia_coli
Degradation_Utilization_Assimilation.Nucleoside_and_Nucleotide_Degradation
Carbohydrate_Biosynthesis.s__Escherichia_coli
Carboxylate_Degradation.s__Escherichia_coli
Carbohydrate_Degradation.s__Escherichia_coli
TCA_cycle.s__Escherichia_coli
Glycolysis.s__Escherichia_coli
Generation_of_Precursor_Metabolites_and_Energy.TCA_cycle
Superpathways.s__Escherichia_coli
WF_Treated
WF_ZnO-free
Feces_7dpw Treated
Feces_14dpw Treated
Feces_14dpw ZnO-free
Feces_0dpw Treated
Feces_0dpw ZnO-free
FD_Treated
Diarrhea_7dpw Treated
Diarrhea_7dpw ZnO-free
0
2
4
LDA SCORE (log 10)

Supplementary Figure S3. Differences in superclass2 grouped pathways and species abundance, returned by LEfSe analysis, most likely explaining the differences among dietary treatments in each sample type and day post weaning.
