# Supplementary material for: Fine-tuning of post-weaning pig microbiome structure and functionality by in-feed zinc oxide and antibiotics use
Source: Front Cell Infect Microbiol. 2024 Feb 7;14:1354449. doi: 10.3389/fcimb.2024.1354449 (PMC10879578; doi:10.3389/fcimb.2024.1354449)
Supplement: Supplementary file 4 [file Table_1.pdf]

Supplementary Table S1. Analysis of the Influence of different factors on the ordination of samples determined using the envfit function of Vegan.

Factor	Species		Pathways	
	R^2	p.val	R^2	p.val
Farm	0.035	0.999	0.055	0.999
Type	0.672	0.001	0.583	0.001
Dpw	0.256	0.001	0.330	0.001
Treatment	0.004	0.786	0.003	0.821
Type_dpw	0.762	0.001	0.610	0.001
Type_dpw_treatment	0.789	0.001	0.674	0.001
