# Supplementary material for: Fine-tuning of post-weaning pig microbiome structure and functionality by in-feed zinc oxide and antibiotics use
Source: Front Cell Infect Microbiol. 2024 Feb 7;14:1354449. doi: 10.3389/fcimb.2024.1354449 (PMC10879578; doi:10.3389/fcimb.2024.1354449)
Supplement: Supplementary file 5 [file Table_2.pdf]

Supplementary Table S2. Results of the permutation multivariate analysis of variance (PERMANOVA) test performed in the ordination analysis.

Factor/Levels	Species		Pathways	
	R^2	p.val	R^2	p.val
Treatment	0.010	0.294	0.014	0.324
Type_dpw	0.560	0.001	0.318	0.001
Feces 0dpw vs Feces 7dpw	0.252	0.002	0.094	0.059
Feces 0dpw vs Feces 14dpw	0.359	0.002	0.156	0.002
Feces 0dpw vs Diarrhea 7dpw	0.405	0.003	0.133	0.052
Feces 0dpw vs WF	0.507	0.002	0.209	0.002
Feces 0dpw vs FD	0.561	0.002	0.272	0.002
WF vs FD	0.065	0.280	0.072	0.117
Feces 7dpw vs Diarrhea 7dpw	0.217	0.009	0.100	0.110
Feces 7dpw vs Feces 14dpw	0.081	0.157	0.066	0.144
Feces 7dpw vs WF	0.495	0.002	0.257	0.002
Feces 7dpw vs FD	0.584	0.002	0.336	0.002
Diarrhea 7dpw vs Feces 14dpw	0.133	0.076	0.121	0.033
Diarrhea 7dpw vs WF	0.377	0.002	0.238	0.002
Diarrhea 7dpw vs FD	0.479	0.003	0.307	0.003
Feces 14dpw vs WF	0.456	0.002	0.273	0.002
Feces 14dpw vs FD	0.560	0.002	0.354	0.002
Type_dpw:Treatment	0.046	0.388	0.086	0.068
Diarrhea 7dpw ZnO-free vs Diarrhea 7dpw Treated	0.480	0.070	0.541	0.110
