# Supplementary material for: Fine-tuning of post-weaning pig microbiome structure and functionality by in-feed zinc oxide and antibiotics use
Source: Front Cell Infect Microbiol. 2024 Feb 7;14:1354449. doi: 10.3389/fcimb.2024.1354449 (PMC10879578; doi:10.3389/fcimb.2024.1354449)
Supplement: Supplementary file 6 [file Table_3.pdf]

Supplementary Table S3. Results of differential abundance analysis of relative abundances between type and dpw levels.

Species	Type and dpw comparison	*P*
Lactobacillus amylovorus	Feces_0dpw vs Feces_7dpw	0.006
	Feces_0dpw vs Feces_14dpw	0.003
Limosilactobacillus reuteri	Feces_0dpw vs Diarrhea_7dpw	0.006
	Feces_0dpw vs Feces_14dpw	0.015
Escherichia coli	Feces_0dpw vs Feces_14dpw	0.006
Prevotella copri	Feces_0dpw vs Feces_7dpw	$P < 0.001$
	Feces_0dpw vs Feces_14dpw	$P < 0.001$
Prevotella sp. CAG:520	Feces_0dpw vs Feces_7dpw	0.043
	Feces_0dpw vs Feces_14dpw	0.037
	Feces_0dpw vs Diarrhea_7dpw	0.001
	Feces_7dpw vs Diarrhea_7dpw	0.033
Anaeromassilibacillus sp. An172	Feces_0dpw vs Feces_7dpw	$P < 0.001$
	Feces_0dpw vs Feces_14dpw	$P < 0.001$
	Feces_0dpw vs Diarrhea_7dpw	$P < 0.001$
Prevotella sp. CAG:873	Feces_0dpw vs Feces_7dpw	$P < 0.001$
	Feces_0dpw vs Feces_14dpw	$P < 0.001$
	Feces_0dpw vs Diarrhea_7dpw	0.001
Catenibacterium mitsuokai	Feces_0dpw vs Feces_7dpw	0.009
	Feces_0dpw vs Feces_14dpw	0.016
Ruminococcus torques	Feces_0dpw vs Feces_7dpw	0.010
	Feces_0dpw vs Feces_14dpw	0.010
	Feces_0dpw vs Diarrhea_7dpw	0.010
Prevotella sp. P3-122	Feces_0dpw vs Feces_7dpw	$P < 0.001$
	Feces_0dpw vs Feces_14dpw	0.004
	Feces_7dpw vs Feces_14dpw	0.037
	Feces_0dpw vs Diarrhea_7dpw	0.037
	Feces_7dpw vs Diarrhea_7dpw	0.037
Methanobrevibacter smithii	Feces_0dpw vs Feces_7dpw	0.018
	Feces_0dpw vs Feces_14dpw	0.006
	Feces_0dpw vs Diarrhea_7dpw	0.001
Lactobacillus johnsonii	Feces_0dpw vs Feces_7dpw	0.034
Phocaeicola vulgatus	Feces_0dpw vs Feces_7dpw	$P < 0.001$
	Feces_0dpw vs Feces_14dpw	$P < 0.001$
	Feces_0dpw vs Diarrhea_7dpw	$P < 0.001$
Blautia obeum	Feces_0dpw vs Feces_7dpw	0.015
	Feces_0dpw vs Feces_14dpw	0.009
